# Zero-Power, High-Frequency Floating Memristor Emulator Circuit and Its Applications

**DOI:** 10.3390/mi16030269

**Published:** 2025-02-26

**Authors:** Imen Barraj, Amel Neifar, Hassen Mestiri, Mohamed Masmoudi

**Affiliations:** 1 Department of Computer Engineering, College of Computer Engineering and Sciences, Prince Sattam Bin Abdulaziz University, Al-Kharj 11942, Saudi Arabia; 2 Systems Integration & Emerging Energies (SI2E), Electrical Engineering Department, National Engineering School of Sfax, University of Sfax, Sfax 3038, Tunisia

**Keywords:** high frequency, memristor emulator, pinched hysteresis loop, floating, MOSFET

## Abstract

This paper presents a novel passive floating memristor emulator that operates without an external DC bias, leveraging the DTMOS technique. The design comprises only four MOSFETs and eliminates the need for external capacitors. The emulator achieves a high operating frequency of around 250 MHz and consumes zero static power. A comprehensive analysis and simulation, conducted using 180 nm CMOS technology, validates the circuit’s performance. The versatility and effectiveness of the proposed emulator are demonstrated through its application in various circuits, including logic gates, a ring oscillator, and analog filters, highlighting its potential for diverse low-power, high-frequency applications. The proposed emulator provides a compact, efficient, and integrable solution for nanoelectronic circuit designs.

## 1. Introduction

Memristors, theorized by Leon Chua in 1971, represent the fourth fundamental passive circuit element, alongside resistors, capacitors, and inductors. These two-terminal devices maintain a relationship between the time integrals of current and voltage, exhibiting a unique memory-like resistance dependent on the history of applied electrical stimuli [1]. This inherent memory effect makes memristors promising candidates for a wide range of applications, including non-volatile memory [2,3], neuromorphic computing [4,5], analog signal processing [6,7], and chaotic circuits [8]. In non-volatile memory, memristors can be used to create storage devices that retain data even when the power is turned off, such as in solid-state drives (SSDs) and flash memory. Similarly, in neuromorphic computing, memristors emulate synaptic functions, enabling the development of brain-like computing systems that can learn and adapt over time, akin to artificial intelligence applications like image and speech recognition. Memristors are also utilized in analog signal processing to process continuous signals in real-time, which is crucial for applications such as audio and video processing, telecommunications, and sensor data analysis. Additionally, memristors enable the design of chaotic circuits, which exhibit complex, unpredictable behaviors, which are valuable in secure communications, random number generation, and advanced cryptographic systems. This versatility highlights the potential of memristors to revolutionize multiple technological domains.

The inherent challenges in fabricating nanoscale memristors motivate the exploration of memristor emulators. While memristors hold immense potential for revolutionizing electronics circuits design, their physical realization, particularly at dimensions compatible with modern integrated circuits, presents significant fabrication hurdles [9,10]. These challenges include several key issues, including limitations in scalability, device-to-device performance variability, and the need for precise control over material properties at the nanoscale. The complex interplay of material properties and the intricacies of nanoscale fabrication processes pose significant obstacles. These challenges hinder the widespread adoption and commercialization of memristors. For example, in metal-oxide memristors, the controlled formation and rupture of conductive filaments are crucial for achieving reliable and predictable switching behavior [6]. Variations in filament formation, caused by material defects and local electric fields, result in significant device-to-device variability. This variability manifests in fluctuations in critical device parameters, including switching voltage and on/off resistance ratios, impacting circuit design and overall performance. Similarly, perovskite-based memristors, despite their promising characteristics [11], face challenges related to ionic migration and material degradation, which can compromise their long-term stability and operational lifespan. These material-specific challenges underscore the critical need for continued research and development in memristor fabrication techniques [6]. Consequently, the pursuit of memristor emulator circuits has gained significant attention. These emulators, constructed using readily available components like transistors and operational amplifiers [12,13,14,15,16,17,18,19,20], offer a practical alternative for investigating memristor dynamics and exploring their potential applications without the constraints imposed by current fabrication limitations. By accurately mimicking the behavior of ideal memristors, emulator circuits provide a valuable solution for circuit designers and researchers to study memristive phenomena, develop novel applications, and accelerate the integration of memristors into future electronics systems.

Several approaches have been proposed for designing memristor emulators, often employing operational transconductance amplifiers (OTAs), current conveyors, and other active components [12,13,14,15,16]. For instance, OTAs are utilized to achieve precise control over current flow, which is essential for accurately replicating the memristive behavior. Current conveyors, such as the second-generation current conveyor (CCII), are favored for their high-speed signal processing capabilities, making them suitable for applications requiring rapid response times [17]. However, many of these circuits suffer from limitations such as high power consumption, limited frequency response, or restrictions in their mode of operation (e.g., grounded configurations). The power consumption of active components can be a significant drawback, especially in large-scale neuromorphic systems where energy efficiency is crucial. For example, in [18], a floating-type memristor emulator using active building blocks demonstrated stable operation at frequencies up to 40 MHz, but the power consumption remained a challenge. In neuromorphic computing applications, where thousands of memristor emulators might be used to mimic neural networks, high power consumption can lead to excessive heat generation and energy costs [19]. Furthermore, the limited frequency response of some emulators restricts their applicability in high-speed signal processing applications. In telecommunications, where signals need to be processed at very high frequencies, the limited bandwidth of some memristor emulators can be a bottleneck. The study in [20] highlighted that while their proposed emulator circuits could handle analog applications, the frequency response was limited, affecting their performance in high-speed environments. The complexity of some designs, requiring multiple active and passive components, can also hinder their practical implementation and scalability. This complexity not only increases the cost and size of the circuits but also makes them more prone to errors and difficult to integrate into larger systems.

The need for simpler, more efficient, and higher-frequency memristor emulators remains a significant challenge, particularly for emerging applications in neuromorphic computing and high-speed signal processing. Researchers are actively exploring new circuit designs to overcome these limitations. Recently, passive memristor emulators, using fewer active components, have been investigated for their potential to reduce power consumption and complexity. For example, in [21], a floating memristor emulator using three NMOS transistors, a grounded capacitor, and a current source has been introduced. While the current source allows for some control over the emulator’s behavior, the maximum operating frequency is limited to 13 MHz. Similarly, the authors in [22] have developed a grounded memristor operating up to 100 MHz using three MOS transistors and a grounded MOS capacitor. However, this design lacks post-fabrication tunability, limiting its adaptability to different applications. Furthermore, the authors did not demonstrate the practical application of their circuit. A recent study demonstrated a passive memristor emulator using the SMIC 130 nm CMOS process, which maintained significant hysteresis curves at input frequencies up to 300 MHz, showing promise for high-speed applications [23].

Furthermore, numerous efforts have been made to develop MOS-based memristor emulators, but many existing designs face limitations related to operating frequency, tunability, and circuit complexity. These limitations often arise from large capacitors, which restrict frequency response and occupy significant chip area. Grounded memristor designs operating in the MHz range, such as those described in [24,25], employ only two MOS transistors and no external capacitors. While this simplifies the circuit, it also sacrifices tunability, a crucial feature for emulating diverse memristive behaviors. In [26], a grounded memristor with seven transistors and a grounded capacitor has been proposed, achieving an operating frequency of 50 MHz. However, the use of a conventional metal plate capacitor limits the potential for further frequency improvements. A subsequent design [27] employed four transistors and a grounded capacitor, enabling post-fabrication memristance tuning but limiting the operating frequency to 10 MHz. These studies suggest that grounded memristor emulators may not be suitable for complex circuits. Floating memristor emulators have also faced challenges in achieving high operating frequencies. Shi et al. designed a floating memristor circuit using three BJTs, two floating resistors, and a floating capacitor, but the maximum operating frequency reached only 20 kHz [28]. Guo et al. presented a floating memristor operating up to 50 kHz using two floating resistors, two diodes, a floating capacitor, and a floating inductor. However, the inclusion of a passive inductor makes the circuit bulky and expensive [29]. A subsequent design by Guo et al. employed two floating resistors, two diodes, and a floating capacitor, allowing for ON/OFF window modulation by varying the input current. However, the large floating capacitor limits the operating frequency to 150 kHz [30]. Furthermore, floating capacitors are generally undesirable for integrated circuit fabrication due to their large size and potential for parasitic effects. These examples illustrate the various trade-offs encountered in designing MOS-based memristor emulators. The limitations observed in existing designs, including low operating frequencies, lack of tunability, grounded operation modes, bulky passive components, large chip areas, and the need for floating capacitors and diodes, motivate the search for improved emulator circuits.

This work addresses these challenges by presenting a novel zero-power, high-frequency floating memristor emulator circuit based only on MOSFET transistors. The proposed design leverages the inherent non-linear characteristics of MOSFETs to emulate memristive behavior without additional active components, minimizing power consumption. The absence of active components also contributes to a higher frequency response, making the emulator suitable for demanding signal processing applications. The floating nature of the emulator further enhances its versatility and applicability in various circuit configurations, allowing for greater flexibility in circuit design. The design utilizes a minimal number of components, simplifying its implementation and potential for integration into larger systems.

The primary objectives of this research are to present the design and operating principle of the proposed zero-power, high-frequency floating memristor emulator; characterize its performance through detailed simulations, validating its memristive behavior and frequency response; and demonstrate its potential applications in specific circuits, such as oscillators and nonlinear filters, highlighting its advantages over existing emulator designs.

The remainder of this paper is organized as follows: Section 2 provides a detailed description of the proposed memristor emulator circuit, including its schematic diagram and operating principle. Section 3 presents the simulation, validating the performance of the emulator and demonstrating its memristive behavior. Section 4 discusses and compares the findings. Section 5 explores the application of the emulator in signal-processing circuits. Finally, Section 6 concludes the paper, summarizing the key findings and discussing future work.

## 2. Proposed CMOS Memristor Emulator

**Figure 1:**
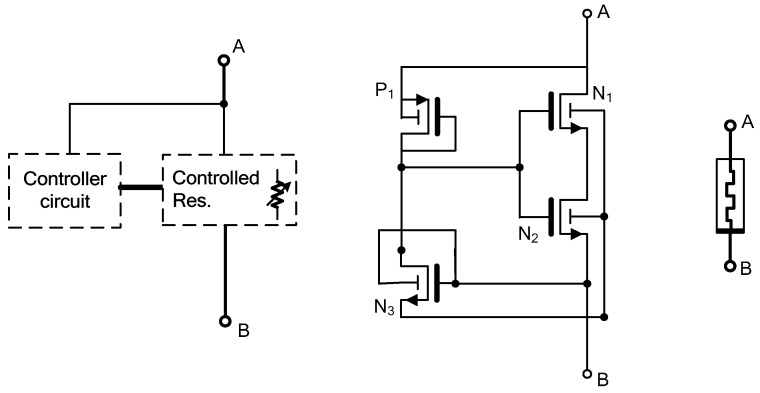
Proposed floating memristor emulator: General block diagram depiction, MOSFET circuit implementation, and its equivalent circuit.

The proposed floating memristor emulator (FMRE) architecture is depicted in Figure 1 and comprises two key components: a controller circuit and a controlled resistor. The controller circuit generates voltage signals to mimic memristor behavior, while the controlled resistor adjusts its resistance based on these signals. The design includes four MOSFET transistors: three NMOS transistors (N1, N2, and N3) and one PMOS transistor (P1), as illustrated in Figure 1. P1 and N3 function as the controller circuit, while N1 and N2 serve as the controlled/variable resistors within the architecture. Unlike many existing memristor emulators, this design eliminates the need for external biasing components, resulting in zero static power dissipation. This feature is particularly advantageous for low-power applications. The input ports, denoted as A and B in Figure 1, provide the interface for integrating the FMRE into circuits. The absence of external biasing requirements simplifies the integration process and reduces the overall circuit complexity.

The proposed FMRE leverages a unique configuration of MOSFETs to achieve its desired characteristics. Transistors P1 and N3 operate in the saturation region, leveraging their high transconductance and non-linear current-voltage characteristics to replicate memristor behavior. In the saturation region, P1 and N3 function as voltage-controlled current sources, enabling precise control over the current flow through the circuit. This mode of operation is particularly advantageous. It ensures high gain and fast response times, both critical for achieving high-frequency performance. Additionally, the saturation region allows P1 and N3 to generate the necessary control signals with minimal distortion, which is vital for maintaining the integrity of the memristive hysteresis loop.

In contrast, transistor N2 operates in the linear region, where it acts as variable resistor. This mode of operation is chosen because it provides a wide and tunable range of resistance values, which is fundamental to emulating the variable resistance behavior of a memristor. The linear region enables N2 to dynamically adjust its resistance with gate voltage changes, accurately mimicking the pinched hysteresis loop of memristors. The drain of N3 is connected to the drain of P1; the voltage at this node (F) supplies the control voltage to the gates of N1 and N2. The source terminal of N3 supplies the control voltage to the body terminals of N1 and N2, both N-type transistors. This substrate control mechanism enhances the tuning of the threshold voltage of N1 and N2 through the body effect, allowing for finer adjustment of their resistance and improving the overall precision of the emulator. This interconnected arrangement enables precise control over the conductance of the controlled resistor, mimicking the variable resistance behavior of a memristor.

The DTMOS technique employed here significantly enhances the circuit’s performance. For optimal low-voltage performance, a MOSFET requires a high Vth at V_GS_ = 0 to minimize leakage and a low Vth at V_GS_ = V_DD_ for high speed. The DTMOS addresses these constraints by connecting the gate and body terminals of a MOSFET. This configuration enables the transistor to exhibit a high threshold voltage when it is off, minimizing leakage, and a low threshold voltage under low voltage supplies, driving high current. Additionally, it features a steeper subthreshold swing and higher carrier mobility compared to a conventional MOSFET. The dynamic body bias voltage makes Vth a function of the input signal. With an input signal applied at the gate terminal, the body bias changes dynamically, maintaining V_GS_ = V_BS_ as the gate and body terminals are shorted together [31]. When the gate input of a MOSFET increases, the source–body junction gets slightly forward-biased, causing Vth to decrease due to the body effect. The dynamic body bias allows both the gate and body terminals to control the potential in the channel region, resulting in high transconductance and faster current flow into the channel. In the proposed FMRE, the DTMOS transistor is essential for achieving memristor behavior at different operating frequencies, thus aiding in reaching the maximum operating frequency of the emulator. The DTMOS technique also ensures efficient operation at low voltages, contributing to the zero static power consumption of the emulator.

Additionally, the internal MOSFET capacitances play a significant role in generating the hysteresis loop. These capacitances introduce a temporal delay between the applied voltage and the resulting current, which is essential for creating the pinched hysteresis behavior characteristic of memristors.

**Figure 2:**
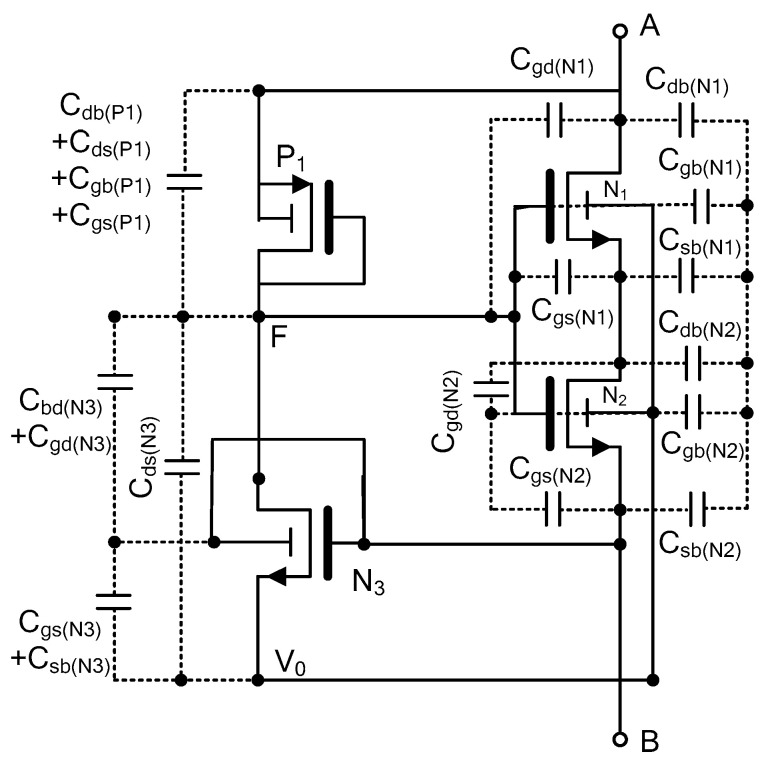
The equivalent FMRE circuit along with internal parasitic capacitors.

The primary internal capacitances to be considered are drain-to-body (C_DB_), source-to-body (C_SB_), drain-to-body (C_SD_), gate-to-source (C_GS_), drain-to-source (C_DS_), and gate-to-drain (C_GD_). The detailed memristor configuration, which includes all these parasitic elements, is illustrated in Figure 2. These capacitances can lead to deviations from ideal memristance values, affecting the rate of memristance change and reducing the pinched hysteresis loop area. However, these effects can be mitigated. By precisely adjusting the aspect ratios of transistors N1, N2, N3, and P1, we can fine-tune the memristance and gain bandwidth, thereby minimizing the impact of parasitic capacitances and achieving the desired performance characteristics.

The theoretical analysis, which incorporates the aforementioned capacitances, has been performed to validate the functionality of the memristor.

**Figure 3:**
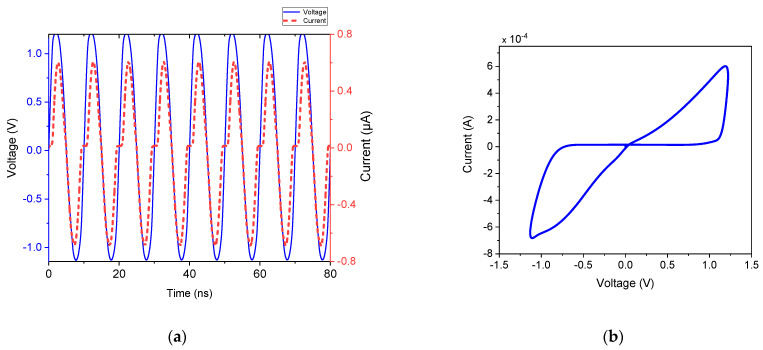
(**a**) Transient curve, and (**b**) V–I characteristic at 100 MHz input.

In Figure 3, considering node A at $\left(v_{in}(t)\right)$ and node B at $\left(yv_{in}(t)\right)$, 0≤y<1. The first step is to derive the voltage $V_{0}(t)$ used for controlling the body voltage of N1 and N2 transistors. Considering the internal capacitors as shown in Figure 2, the nodal current equation at the node $S_{N3}$ can be expressed as follows:

(1) $$C_{gs(N3)}\;\frac{d\left(yV_{in}\left(t\right)-V_{0}(t)\right)}{dt}+C_{DS(N3)}\;\frac{d\left(V_{F}\left(t\right)-V_{0}(t)\right)}{dt}≅\;K_{N3\;}{\left(V_{SGN3}-V_{THN3}\right)}^{2}\;$$

where the transconductance parameters are $K_{N3\;}=\frac{1}{2}{µ}_{n}C_{ox}{\left(\frac{w}{L}\right)}_{N3}$, ${\left(\frac{w}{L}\right)}_{N3}$ is the aspect ratio of N3, and $V_{SGN3}$ is the source-to-gate voltage of N3. Considering $C_{e1}\approx C_{e2}=C_{0}$, where $C_{e1}=C_{gd(N1)}+C_{gb(P1)}+C_{db(P1)}+C_{ds(P1)}+C_{gs(P1)}$, and $C_{e2}=C_{gd(N3)}+C_{db(N3)}$, using voltage analysis, the voltage at the node F, $V_{F}\left(t\right)$, can be expressed as $\frac{\left(y+1\right)}{2}C_{0}V_{in}(t)$. The obtained equation is similar to the one developed in [32]. A similar analytical approach is used to derive the expression for the emulator memductance. We consider $N=C_{gs(N3)}/C_{DS(N3)}$, where we can take it greater than 1, as gate–drain capacitance is often an order of magnitude smaller than the gate–source capacitance. Since the body of the N_3_ is connected to the gate, we take $V_{THN3}=V_{THN3\;(0)}+\gamma \left(\sqrt{\left|2\psi_{s}+V_{SBN3}\right|}-\sqrt{2\psi_{s}}\right)≅V_{THN}+K_{B}V_{SBN3}$, where by replacing $x\left(t\right)=\left(y+\frac{N\left(y+1\right)C_{0}}{2}\right)V_{in}\left(t\right)-\left(N+1\right)V_{0}\left(t\right),$ and the body constant of NMOS, $K_{B}≅\gamma {\left(2\psi_{s}\right)}^{\frac{1}{2}}/2$. Considering $K^{\prime }=$ $K_{N3}\sqrt{1+K_{B}}$, Equation (1) can be re-arranged as

(2) $$C_{gs(N3)}\;\frac{d\left(x\left(t\right)\right)}{dt}≅\;K_{N3}\sqrt{1+K_{B}}\;{\left(x\left(t\right)-\beta V_{in}\left(t\right)+\left(N+2\right)V_{0}\left(t\right)-\frac{V_{THN}}{1+K_{B}}\right)\;}^{2}$$

where $\beta =2y+\frac{N\left(y+1\right)C_{F}}{2}$, and we can neglect the terms ${\left(-2+\frac{\beta V_{in}\left(t\right)-\left(N+2\right)V_{0}\left(t\right)+\frac{V_{THN}}{1+K_{B}}}{x(t)}\right)}^{2}-\frac{6(N+2)V_{0}(t)}{x(t)}$.

Now, by taking $z_{1}\left(t\right)=x^{-1}(t)$ and $\frac{d\left(z_{1}(t)\right)}{dt}=-\frac{1}{x^{2}(t)}\frac{d(z_{1}(t)}{dt}$, Equation (2) will be equivalent to the following expression:

(3) $$\frac{d\left(z_{1}(t)\right)}{dt}-\frac{6\beta K^{\prime }}{C_{gs(N3)}}\left(V_{in}\left(t\right)+\frac{V_{THN}}{1+K_{B}}\right)z_{1}(t)≅\frac{3K^{\prime }}{C_{gs(N3)}}$$

It is evident that Equation (3) resembles a first-order linear non-homogeneous differential equation. A possible solution for this would be

(4) $$z_{1}\left(t\right)≅De^{\int \frac{6\beta K^{\prime }}{C_{gs(N3)}}\left(V_{in}\left(t\right)+\frac{V_{THN}}{1+K_{B}}\right)dt}+A$$

where A and D are integration constant.

From Equation (4), using binomial expansion and exponential series (neglecting higher order terms), $V_{0}\left(t\right)$ can be written in terms of $z_{1}\left(t\right)$ and $V_{in}\left(t\right)$ as

(5) $$V_{0}\left(t\right)≅D_{1}-\frac{D_{2}K^{\prime }}{C_{gs\left(N3\right)}}\emptyset_{0}\left(t\right)+D_{3}V_{in}\left(t\right)$$

where $D_{1}$, $D_{2}$ and $D_{3}$ are integration constant.

The two transistors, N1 and N2, are connected in series, with their gates and bulk controlled by the same voltage. Therefore, N1 and N2 can be replaced by a single transistor (N) with the same length, but with a width equal to the sum of the widths of N1 and N2. The gate of this equivalent transistor will be connected to node F, the drain to node A, and the source to node B. Given that $W_{N2}=\alpha W_{N1}$, where α>1, the equivalent transistor will have a width of $\left(1+\alpha \right)W_{N1}$.

The corresponding memductance of the proposed floating memristor, W(Ø(t)), can be derived from the current of the equivalent transistor as

(6) $$I_{in}\left(t\right)=K_{N}\left(V_{GS(N)}-V_{TH(N)}\right)V_{in}(t)$$

where $K_{N}={µ}_{n}C_{ox}\left(\frac{\left(1+\alpha \right)W_{N1}}{L}\right)$, and $V_{GS(N)}=V_{F\left(t\right)}-yV_{in}(t)$. From the circuit in Figure 2 considering $C_{e3}=C_{sb(N2)}+C_{sb(N3)}+C_{gs(N3)}$, using voltage analysis, the voltage $V_{F\left(t\right)}$ can be expressed as $\left(\frac{C_{e2}+C_{e3}}{C_{e3}}yV_{in}\left(t\right)-\frac{C_{e2}}{C_{e3}}V_{0}\left(t\right)\right)$. Thus Equation (6) can be re-arranged as

(7) $$W\left(\emptyset (t)\right)=\frac{I_{in}\left(t\right)}{V_{in}(t)}=K_{N}\left(E_{1}V_{in}\left(t\right)+\frac{E_{2}K^{\prime }\emptyset_{0}\left(t\right)}{C_{gs\left(N3\right)}}-E_{3}+Vs\right)$$

where $E_{1}=$ $\frac{C_{e3}+C_{e4}}{C_{e4}}-1+D_{3}$, $E_{2}=\frac{C_{e2}+C_{e3}}{C_{e3}}$, and $E_{3}=\frac{C_{e2}\;D_{4}}{C_{e3}}$; (Vs) is the additional voltage caused by intrinsic capacitances, body effect, transistor mismatch, internal noise, and parasitic. These effects can be observed during post-layout simulation and measurement results.

## 3. Simulation Results

The proposed FMRE design has been extensively investigated through simulations conducted using Advanced Design System with a 180 nm CMOS technology process. The MOSFET transistor aspect ratios employed in the design are detailed in Table 1. The careful selection and optimization of these aspect ratios are essential for achieving the desired memristor emulation behavior and maximizing the operating frequency of the FMRE.

The functionality of the proposed memristor emulator is demonstrated in Figure 3. Figure 3a illustrates a multiple-cycle transient analysis of the input voltage signal and corresponding current signal of the memristor at 100 MHz. The corresponding FMRE hysteresis curve is depicted in Figure 2. The asymmetrical current waveform effectively demonstrates the memristor emulator’s nonlinear behavior. This behavior arises from the inherent nonlinear characteristics of the MOSFETs and the presence of parasitic elements within the circuit [33,34]. These parasitic elements significantly impact the overall performance and behavior of the memristor emulator. Consequently, the waveform deviates from the ideal symmetrical shape, showcasing the complexities involved in achieving accurate memristor emulation.

The pinched hysteresis loop results from a temporal offset between the peak and trough values of current and voltage, which contrasts with the typical symmetrical bow-tie curve characteristic of memristive behavior. Despite its asymmetry, the hysteresis loop in the proposed design allows for clear differentiation between high and low resistance states, which are controllable through the application of appropriate voltage polarity.

**Figure 4:**
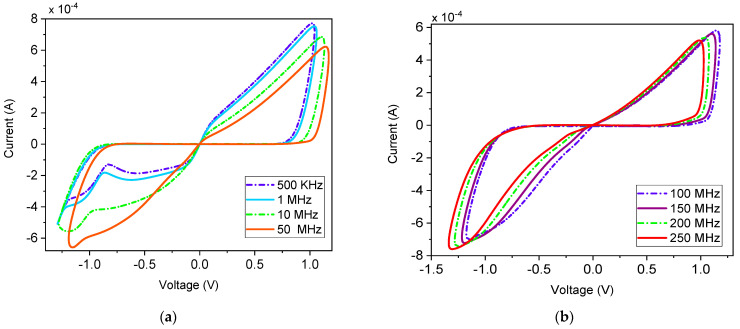
(**a**) Transient curve, and (**b**) V–I characteristic at 100 MHz input.

The pinch hysteresis loop is a fundamental characteristic of memristors, demonstrating a reduction in current to zero when no voltage is applied. This behavior is essential for understanding memristors’ unique electrical properties. One of memristors’ most distinctive features is the alteration of their hysteresis loop with frequency. Specifically, the loop’s behavior tends to diminish as frequency increases. This frequency-dependent behavior serves as a key identifier, or fingerprint, of memristor devices, setting them apart from other electronic components. This phenomenon is clearly illustrated in Figure 4, where the pinch hysteresis loop remains consistent across frequencies from 500 kHz to 250 MHz. Such consistency across a wide frequency spectrum underscores the robustness and reliability of the proposed FMRE. The ability of memristors to maintain their pinch hysteresis loop at different frequencies is crucial for their performance in dynamic environments, where operational frequencies can vary significantly.

### 3.1. Effect of Temperature on the Pinched Hysteresis Loop

**Figure 5:**
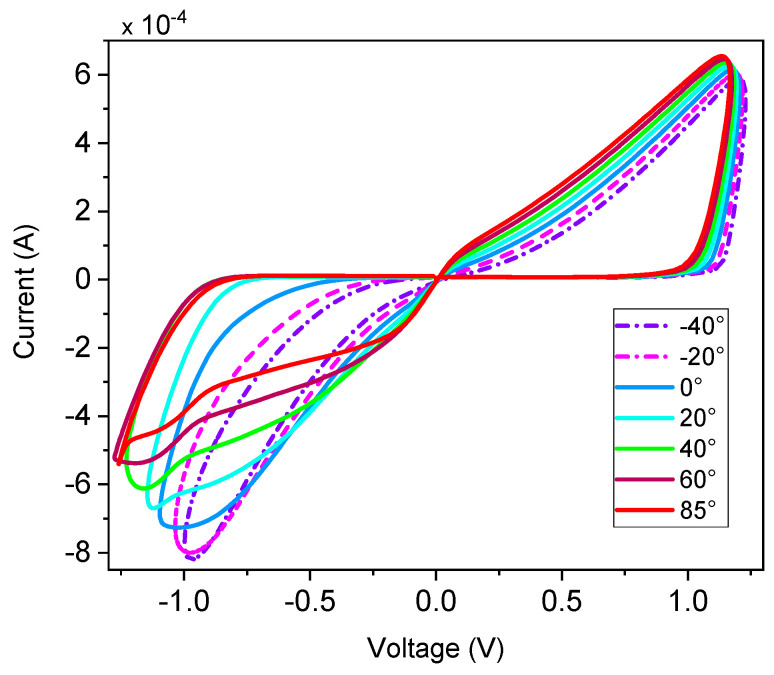
V–I curve for 50 MHz input at different temperatures.

The proposed memristor circuit exhibits a temperature-dependent current behavior, as shown in Figure 5. This figure depicts the effect of temperature variations from −40 °C to +85 °C on the memristor hysteresis loop at a frequency of 50 MHz. The results indicate a positive correlation between temperature and current, with the memristor current increasing as the temperature rises. Despite these temperature-induced changes, the fundamental characteristics, or “fingerprints,” of the memristor remain unaltered. Furthermore, Figure 5 presents the pinch hysteresis loop for the industrial temperature range of −40 °C to +85 °C, demonstrating that the proposed memristor consistently exhibits this behavior across the entire considered temperature range.

### 3.2. Process Variations

**Figure 6:**
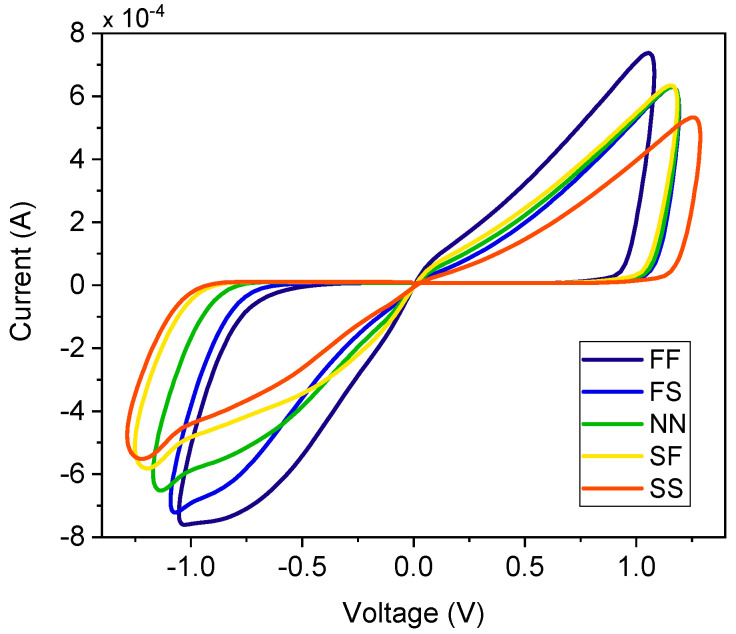
V–I curve at different process corners for 50 MHz input.

Process variations inherent in the monolithic integration significantly impact circuit performance. Therefore, a comprehensive corner analysis is essential to evaluate the robustness of the proposed FMRE. This work examines the proposed emulator circuit across various process corners, including slow-slow (SS), slow-fast (SF), nominal-nominal (NN), fast-slow (FS), and fast-fast (FF). Figure 6 depicts the voltage–current characteristics across these corners. As illustrated in Figure 6, the memristor current in SS mode is notably lower than that in FF mode. This observation is consistent with the expected impact of process variations on transistor performance. Slower process corners typically result in reduced current drive capability, while faster corners exhibit increased current flow. At an input signal frequency of 50 MHz, the nominal current is 0.64 mA. Table 2 presents the percentage deviation in current across various process corners. Also, the proposed memristor circuit maintains a pinched hysteresis loop across all process corners, demonstrating its robust operation despite variations in device parameters. Although minor variations in the hysteresis loop curve are observed, the pinched characteristic crucial for memristive behavior is consistently preserved. This analysis demonstrates the circuit’s predictable and consistent response to process variations, a critical factor for reliable operation in real-world applications.

### 3.3. Voltage Variations

**Figure 7:**
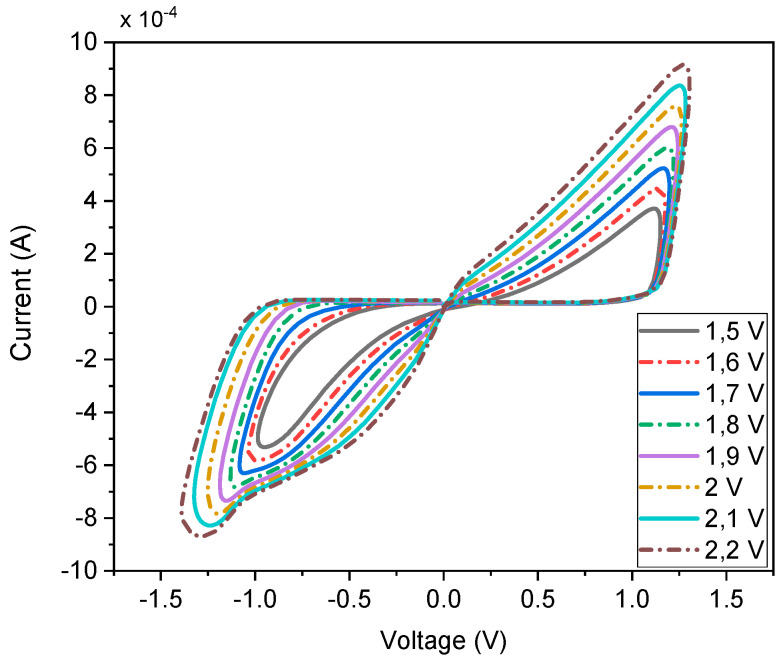
V–I curve at different input signal amplitudes for 50 MHz input.

While the proposed emulator operates without an external DC bias, we have analyzed the emulator’s performance under varying input signal amplitudes, ranging from 1.5 V to 2.2 V. The simulation results, illustrated in Figure 7, demonstrate that the emulator maintains consistent memristive behavior across this voltage range. These results highlight the emulator’s adaptability to varying voltage conditions, ensuring reliable performance in practical applications where input signal amplitudes may fluctuate

### 3.4. Nonvolatility Test

**Figure 8:**
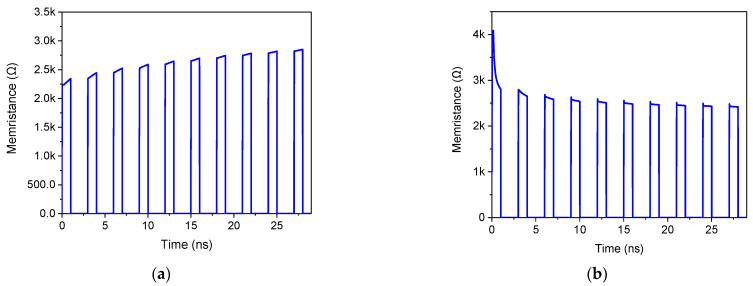
Nonvolatility test of the proposed FMRE. (**a**) Incremental mode, and (**b**) decremental mode.

The non-volatility of the proposed floating memristor emulator, a critical characteristic for memory applications, is investigated. To assess this property, a train of pulses with a period of 3 ns, a pulse width of 1 ns, and an amplitude of 1.8 V was applied to the input terminal. Figure 8 illustrates the variation in memristance for both incremental and decremental configurations of a memristor. The results demonstrate a distinct change in memristance correlated with the applied input pulses.

For the incremental configuration, when the input voltage is high (during the 1 ns pulse duration), the memristance increases. As shown in Figure 8a, during the first pulse period, the memristance rises from 2.240 kΩ to 2.34 kΩ. Crucially, during the subsequent low input voltage period (the remaining 2 ns of the 3 ns cycle), the memristance remains stable at the increased value, demonstrating the non-volatile nature of the emulator. This behavior repeats in subsequent cycles, with the memristance increasing further during high input voltage periods and holding its value during low input voltage periods. For example, during the second pulse period, the memristance increases from 2.34 kΩ to 2.44 kΩ and then holds this value until the next high input voltage pulse. For the decremental configuration, when the input voltage is high, the memristance decreases. As depicted in Figure 8b, during the first pulse period, the memristance decreases from 4.08 kΩ to 2.88 kΩ. Crucially, during the subsequent low input voltage period, the memristance remains stable at the reduced value, demonstrating the non-volatile nature of the emulator. This behavior repeats in subsequent cycles, with the memristance decreasing further during high input voltage periods and holding its value during low input voltage periods. For example, during the second pulse period, the memristance decreases from 2.88 kΩ to 2.65 kΩ and then holds this value until the next high input voltage pulse.

This consistent pattern of memristance change and retention confirms the non-volatile memory characteristic of the proposed circuit. This non-volatile behavior is a key enabler for various applications, including non-volatile memory arrays and neuromorphic computing, where the ability to retain state information without a continuous power supply is essential.

### 3.5. Series and Parallel Combination of Memristors

**Figure 9:**
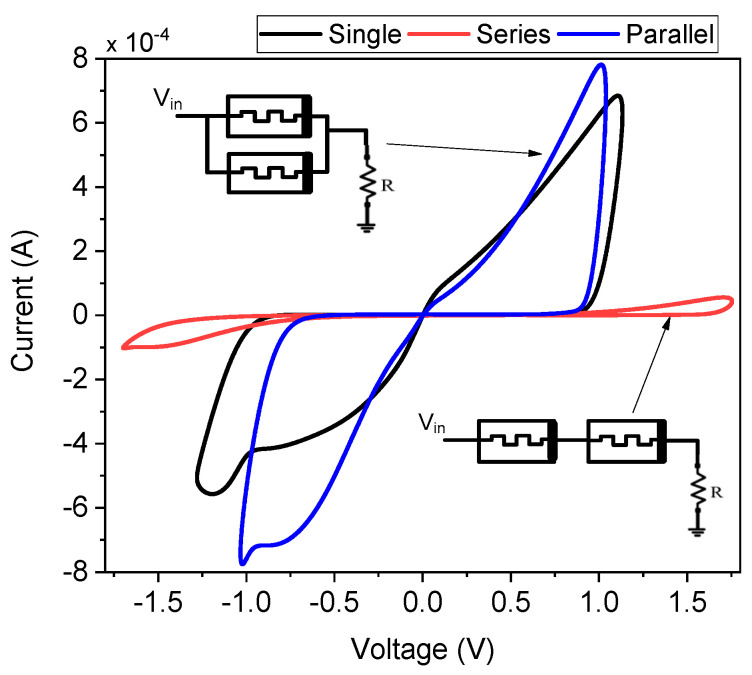
V–I curve for different combinations of memristor at 10 MHz.

The proposed FMRE showcases its adaptability through the analysis of different setups, such as series and parallel connections. Figure 9 presents the voltage–current characteristics for single, parallel, and series arrangements of the emulator. As expected, and clearly illustrated in Figure 9, the parallel configuration exhibits the highest current flow, while the series configuration yields the lowest. This behavior aligns with fundamental circuit principles, where parallel resistances offer a lower equivalent resistance and, thus, a higher current path, whereas series resistances increase the equivalent resistance, thereby reducing current flow for a given applied voltage. The observed V–I characteristics in each configuration confirm the predictable and consistent behavior of the emulator when integrated into more complex circuit topologies. This adaptability is crucial for practical applications, where memristor emulators are often employed as building blocks within larger circuits and systems. The ability to operate effectively in both series and parallel arrangements expands the potential applications of the proposed emulator, including its use in memristor crossbar arrays, neuromorphic circuits, and other emerging technologies. Further investigation into the behavior of these configurations under varying frequencies and input signals could provide valuable insights for optimizing performance in specific application scenarios.

### 3.6. Monto Carlo Simulation

**Figure 10:**
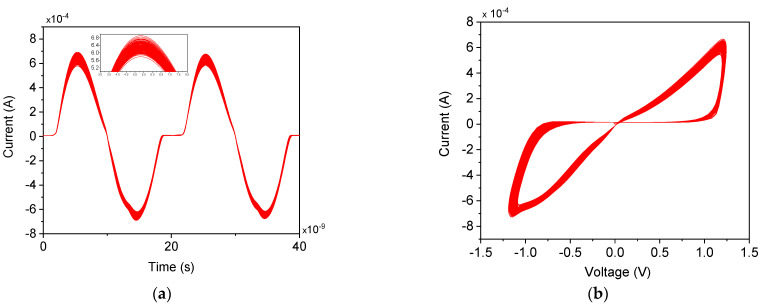
(**a**) Transient curve and (**b**) V–I characteristic at 100 MHz input.

The current of the designed memristor may vary from the intended value due to changes in transistor parameters. Monte Carlo analysis is a valuable technique for determining the impact on memristor current due to drift of parameters of transistors. Therefore, a Monte Carlo analysis, taking into account variations in process and mismatch parameters, was conducted to demonstrate the potential fabrication variation of the proposed FMRE. These responses were obtained by running Monte Carlo simulations 200 times, considering a ±5% uniform deviation in transistor threshold voltages and aspect ratios. The analysis of the memristor circuit in Figure 10 reveals that the deviation in memristor current is limited to ±65 µA, representing only an 11% variance from the nominal value of 0.6 mA. This demonstrates the stability of the circuit design even when components exhibit slight variations within tolerance limits. It is crucial to emphasize that despite these variations, the memristor operates within acceptable limits, ensuring the overall stability and reliability of the circuit under varying process conditions. This highlights the robustness of the design and its ability to maintain consistent performance despite minor component discrepancies.

## 4. Comparison and Discussion

The memristor emulator circuit proposed in this study operates at a frequency of 250 MHz using a 0.18 µm technology process. It is designed with a floating configuration and utilizes only four transistors.

This section aims to highlight the advantageous features of the proposed memristor circuit compared to other existing emulators. The performances of the state-of-the-art emulators and the presented model are summarized in Table 3, detailing various aspects such as circuit structure, technology, operating frequency, and power consumption. Below are some of the key benefits of the proposed work.

- Simplified architecture: The architecture of the proposed model is notably simpler, utilizing fewer transistors compared to the design in [13,14,25,34,35,36,37,38,39,40,41,42]. This reduction in complexity can lead to easier implementation and lower production costs.
- The memristor emulator circuits referenced in [13,14,20,25,30,31,34,39,41,43,44,45,46] incorporate passive components. In contrast, the developed floating memristor is designed without any passive components.
- Zero power consumption: The proposed memristor emulator model operates without any bias, resulting in zero power consumption. This is a significant improvement over other models that require a power supply [20,31,37,39,40,43].
- Enhanced frequency response: Some emulators, as noted in references [14,20,21,23,25,31,40,41,42,43,44,45,46], utilize fewer MOSFETs. However, they typically operate at lower frequencies compared to the proposed circuit. This means that the proposed circuit offers a superior frequency response, making it more suitable for high-frequency applications.
- The proposed circuit is of the floating type, which makes it highly versatile for integration into various circuits, as demonstrated in references [20,30,31,41,42,43,46]. This flexibility allows it to be effectively utilized in a wide range of applications.

These features collectively enhance the proposed memristor emulator circuit’s performance.

**Table 3:** Comparison of the proposed memristor emulator circuit with existing design.

Ref.	No. of MOSFET	No. of Passive Components	Floating/ Grounded	Operating Frequency	Power Consumption	Technology Used
[13]	17	C (1)	Floating	1 MHz	NA	0.18 µm
[14]	9	C (1)	Grounded	2 KHz	NA	45 nm
[20]	3	C (1)	Floating	13 MHz	6.725 µW	0.18 µm
[21]	4	0	Grounded	100 MHz	NA	0.18 µm
[23]	3	0	both	30 MHz	0	0.18 µm
[25]	7	C (1)	Grounded	50 MHz	NA	0.18 µm
[30]	4	C (1)	both	500 MHz	0	0.18 µm
[30]	5	0	both	500 MHz	0	0.18 µm
[31]	4	C (1)	Floating	3 MHz	8.24 µW	0.18 µm
[34]	16	R (2), C (1)	Floating	50 MHz	NA	0.18 µm
[35]	40	R (2), C (1)	Grounded	1 MHz	NA	0.35 µm
[36]	30	R (3), C (1)	both	10 MHz	NA	0.25 µm
[37]	16	C (1)	Floating	Few Hz	8.05 µW	0.18 µm
[38]	24	R (3), C (1)	Grounded	1.7 MHz	NA	0.18 µm
[39]	29	R (1), C (1)	Grounded	26.3 MHz	9.567 µW	0.18 µm
[40]	4	0	Grounded	100 KHz	40 µW	0.18 µm
[44]	3	C (1)	Grounded	100 KHz	0	0.18µm
[41]	6	C (1)	Floating	10 Hz	NA	0.18 µm
[42]	7	0	Floating	1 MHz	NA	0.13 µm
[43]	4	0	Floating	50 MHz	2.6 µW	90 nm
[45]	3	C (1)	Grounded	24 MHz	0	0.18 µm
[46]	1	R (1), C (1)	both	80 MHz	0	45 nm
This work	4	0	Floating	250 MHz	0	0.18 µm

The proposed memristor emulator’s ability to operate at 250 MHz significantly enhances its applicability in a wide range of real-world scenarios, particularly in high-speed and energy-efficient systems. In high-frequency signal processing applications, such as telecommunications and radar systems, the emulator can be seamlessly integrated into analog filters, modulators, and demodulators to improve signal integrity and processing speed, meeting the demands of modern communication technologies like 5G and beyond. For neuromorphic computing applications, the high-frequency operation enables faster synaptic weight updates and signal propagation, reducing latency and enhancing real-time decision making in autonomous vehicles, robotics, and IoT devices. Additionally, the emulator’s zero static power consumption makes it an attractive candidate for energy-efficient computing systems, such as non-volatile memory arrays and low-power logic circuits, which are critical for data centers and mobile devices. Its high-frequency performance also benefits analog and mixed-signal circuits, including oscillators, phase-locked loops (PLLs), and analog-to-digital converters (ADCs), by providing tunable resistance with minimal power overhead. Furthermore, the emulator’s robust operation at 250 MHz opens new possibilities for secure communication systems, such as chaotic encryption and random number generation, where high-frequency, unpredictable signals are essential for ensuring data security. These diverse applications underscore the practical significance of the proposed emulator and its potential to advance both analog and digital circuit design.

## 5. Applications

To validate the effectiveness of the proposed memristor emulator, it is essential to design and test various signal processing circuits. In this study, the functionality of the proposed memristor emulator was evaluated through the design of logic gates, ring oscillators, and filters. By incorporating the memristor emulator into these specific circuits, we were able to thoroughly assess its performance and functionality.

### 5.1. Memristive Logic Gates

**Figure 11:**
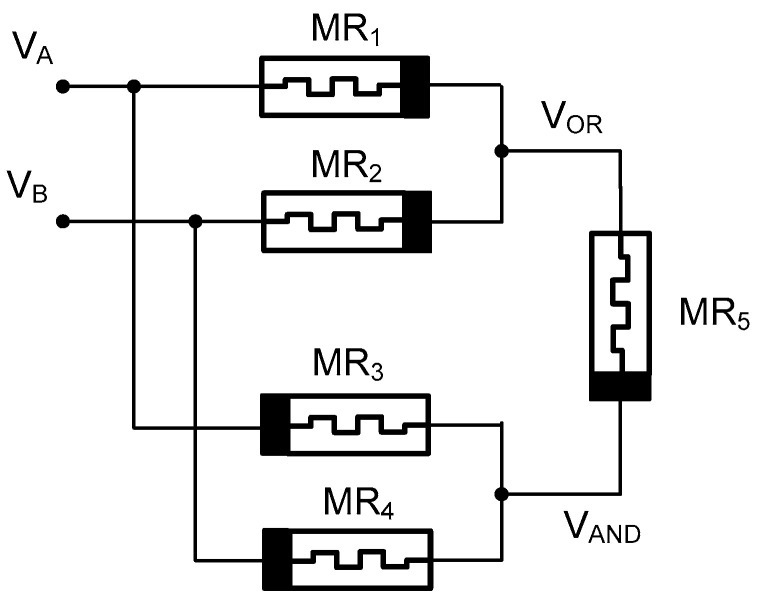
Logical AND, OR, and XOR operations using memristor.

The proposed memristor emulator’s ability to modulate memristance based on current direction enables its integration into fundamental Boolean logic gates, such as AND, OR, and XOR. Figure 11 illustrates the circuit diagrams for memristor-based OR, AND, and XOR gate configurations [39]. In these configurations, the memristance of each device (MR1–5) changes dynamically depending on the applied voltage and current flow. When current flows from high to low potential, the memristance of one device increases to R_OFF_ while the other decreases to R_ON_. This behavior is leveraged to implement logic operations: In the AND gate, the current flow through MR_3_ and MR_4_ establishes distinct memristance values, while in the OR gate, the polarities are reversed to achieve the desired output.

Output voltages V_OR_ and V_AND_ are calculated using the voltage divider rule and can be expressed as follows [40]:

(8) $$v_{OR}=\frac{{RM}_{2}\;v_{A}+{RM}_{1}\;v_{B}}{{RM}_{1}+{RM}_{1}}\;;\;v_{AND}=\frac{{RM}_{4}\;v_{A}+{RM}_{3}\;v_{B}}{{RM}_{3}+{RM}_{4}}\;$$

Furthermore, a memristor-based XOR gate is implemented using AND and OR gates. The XOR gate inputs are the AND gate inputs, while the OR gate inputs serve as control inputs. The initial memristance of MR_5_ is R_OFF_, and its logic value is considered the output. By using a larger memristance value compared to other memristors, MR5 acts as an open circuit, making the XOR gate output voltage equal to $v_{AND}-v_{OR}$ [40]. When one input is logic 1 and the other is 0, current flows from high to low potential through MR_3_, decreasing its memristance to R_ON_ and increasing M4’s memristance to R_OFF_. MR_1_ and MR_2_ have memristances of R_ON_ and R_OFF_, respectively. Using the voltage divider rule, the output V_AND_ is observed as 1, while the output VOR is found to be 0.

**Figure 12:**
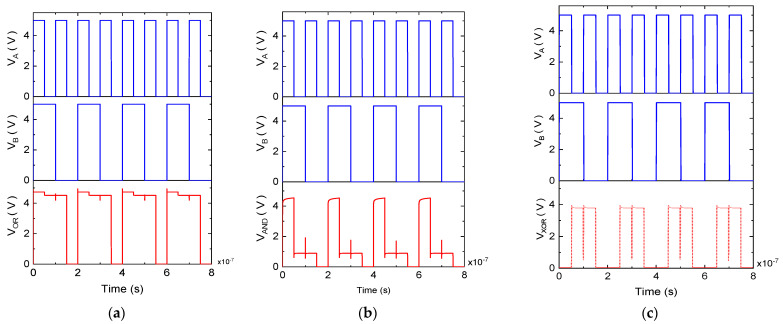
Simulated results of (**a**) OR, (**b**) AND, and (**c**) XOR gates.

Figure 12 presents the simulated input and output waveforms for all three gate configurations, with the VA and VB signals having periods of 100 ms and 200 ms, respectively. During each 50 ms pulse width within the 200 ms output cycle, the gate output signal updates its value based on the two input values. Since the input signal periods are 100 ms and 200 ms, the fundamental period of the output is 200 ms, which is the least common multiple of the input periods.

### 5.2. Memristive Ring Oscillator

**Figure 13:**
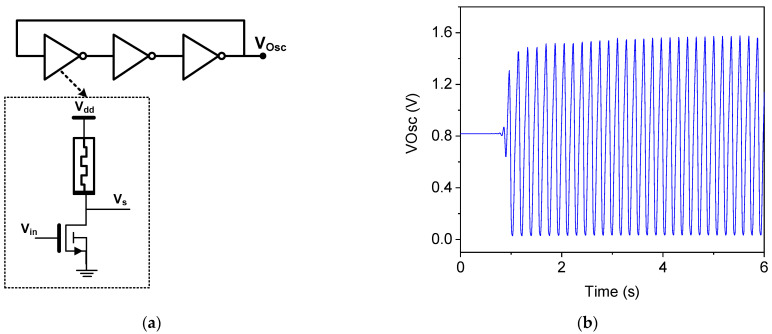
Memristive ring oscillator. (**a**) Circuit and (**b**) output signal.

A ring oscillator is an electronic oscillator that produces a continuous waveform in the form of a ring. It typically consists of an odd number of inverters connected in a loop, with the output frequency determined by the delay through each inverter. The simplicity and reliability of ring oscillators make them a popular choice for various applications in electronics, such as clock signal generation and frequency synthesis. Figure 13a illustrates the memristor-based ring oscillator circuit, which comprises three memristive NMOS inverters. Each stage includes one NMOS transistor and one memristor, with the memristor providing the necessary resistance for oscillation. This configuration leverages the unique properties of memristors to enhance the performance and stability of the oscillator. The three NMOS transistors used in the design have identical dimensions, with a length of 0.18 µm and a width of 20 µm. The simulated output signal of the oscillator, as shown in Figure 13b, demonstrates an oscillation frequency of 5.7 GHz and a startup time of 1ns. This memristor-based ring oscillator provides a compact and efficient solution for generating high-frequency signals. Its straightforward design makes it an appealing choice for a wide range of electronic applications.

### 5.3. Memristive Filters

**Figure 14:**
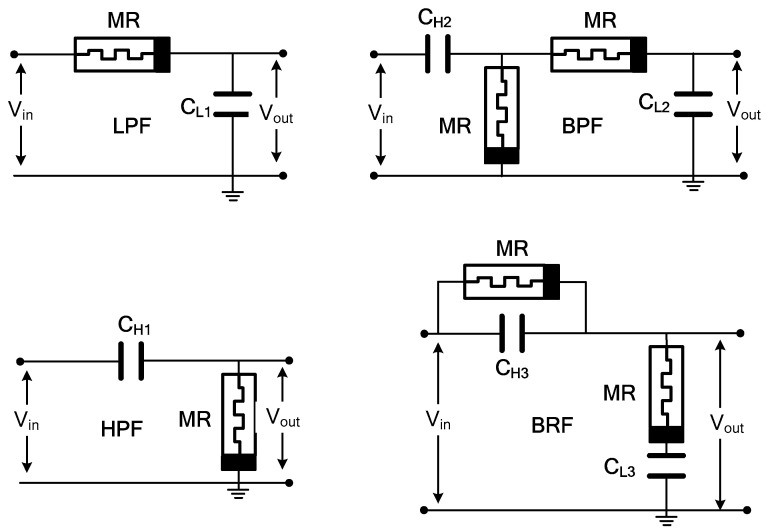
Proposed FMRE application as filters (HPF, LPF, BPF, and BRF circuits).

**Figure 15:**
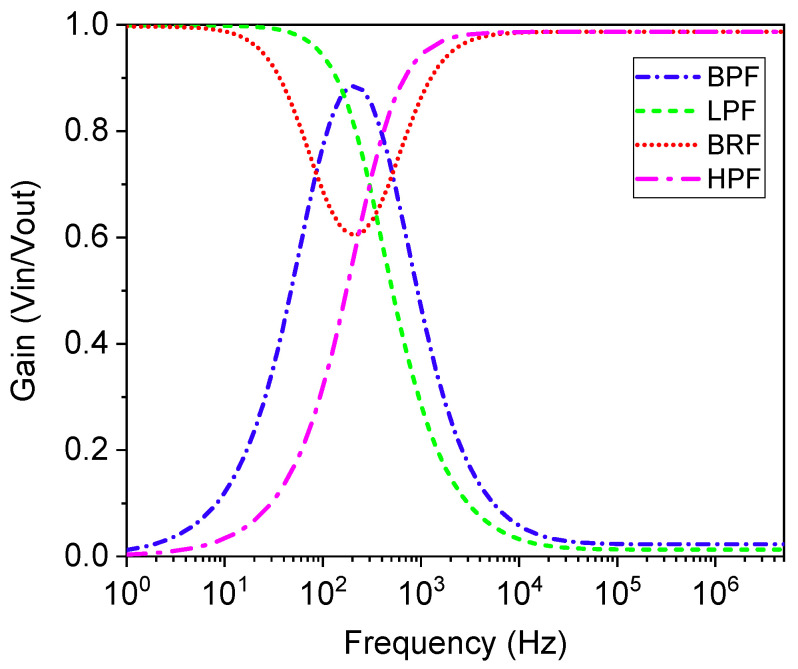
Frequency response for HPF and LPF at C = 1pF, and BPF and BRF at C1 = 2pF & C2 = 1pF.

In traditional filters, a resistor–capacitor (RC) parallel circuit provides a constant cut-off frequency. However, incorporating memristors introduces an adjustable gain characteristic. The variation in the memristance value allows for tuning the filter circuit’s cut-off frequency. To validate the functionality of the proposed memristor emulator, various filter circuits, including low pass filter (LPF), high pass filter (HPF), band pass filter (BPF), and band reject filter (BRF), were designed and simulated. Figure 14 illustrates the circuits for the HPF, LPF, BPF, and BRF, respectively. The frequency responses of these filters are shown in Figure 15. It is important to note that the standard resistor in these circuits has been replaced with the proposed FMRE. According to [30], the cut-off frequency of LPF and HPF filters can be expressed as $f_{c}=1/\left[2\pi C\left(W^{-1}\left(\emptyset (t)\right)\right)\right]$. For the BPF and BRF filters, the low and high cut-off frequencies can be expressed as $f_{cl}=1/\left[2\pi C1\left(W^{-1}\left(\emptyset (t)\right)\right)\right]$ and $f_{ch}=1/\left[2\pi C2\left(W^{-1}\left(\emptyset (t)\right)\right)\right]$ [40].

## 6. Conclusions

This work introduced a novel, zero-power, high-frequency floating memristor emulator circuit implemented using only four MOSFETs. The design eliminates the need for external capacitors, resulting in a compact and efficient circuit. Simulations performed using 180nm CMOS technology validated the FMRE’s performance and its ability to operate across a wide frequency range, around 250 MHz. The efficiency of the proposed FMRE was demonstrated through its successful implementation in various applications, including logic gates, a ring oscillator, and a range of filter circuits.

This research has demonstrated the potential of the proposed zero-power, high-frequency floating memristor emulator circuit through extensive simulation. To further validate and expand upon these promising results, several key areas will be the focus of future work. The FMRE circuit will be fabricated using a suitable CMOS process. This fabrication step will enable experimental verification of the simulated performance characteristics, including power consumption, operating frequency, and dynamic range. Rigorous measurements will be conducted to assess the FMRE’s behavior under various operating conditions and compare these experimental results with the simulation predictions. This empirical validation is crucial for establishing the practical viability of the FMRE. Furthermore, future research will explore the integration of the proposed FMRE into a variety of real-world applications, including in-memory computing and diverse signal processing circuits. This practical implementation will further demonstrate the versatility and effectiveness of the FMRE, showcasing its potential for widespread adoption in both analog and digital circuit design.

## Figures and Tables

**Table 1:** Aspect ratios of the MOSFET used in the design.

MOSFET	Aspect Ratio (W/L) (in µm)
N1	10/0.18
N2	20/0.18
N3	4/4
P1	4/0.18

**Table 2:** Deviation in memristor current for process corner simulation.

Process Corner	FF	FS	SF	SS
Current (mA)	7.41	6.32	6.35	5.31
Variation in current (%)	+18.18	+0.63	+1.27	−15.31

## Data Availability

Data are contained within the article.
